# Comparison of different osteotomy schemes in the repair and treatment of moderate to severe hallux valgus deformities

**DOI:** 10.1097/MD.0000000000041251

**Published:** 2025-02-14

**Authors:** Zhe Xie, Zhaona Wang, Shan Zhai, Jianzhong Wu, Lixing Zhang, Yanbing Gao, Hailong Wu

**Affiliations:** a Department of Orthopaedics (Fang Bei), Shijiazhuang People’s Hospital, China; b Outpatient Operating Room, Shijiazhuang People’s Hospital, China; c Department of Orthopaedics, Shijiazhuang People’s Hospital, China; d Department of Orthopaedics (Fan Xi), Shijiazhuang People’s Hospital, China.

**Keywords:** application effectiveness, corrective treatment, dual-plane osteotomy of the first metatarsal, moderate-to-severe hallux valgus deformity, safety, scarf osteotomy

## Abstract

To explore the application efficacy and safety of different osteotomy schemes in the repair and treatment of moderate to severe hallux valgus deformities. A sum of 117 patients with moderate to severe acute hallux valgus who underwent treatment at our hospital from April 2020 to April 2022 were selected. The envelope method was used to group the patients. The control group (n = 58) underwent double-plane osteotomy of the first metatarsal bone, while the observation group (n = 59) underwent Scarf osteotomy. The clinical efficacy of the 2 groups was compared, the differences in imaging results between the 2 groups at 6 months after surgery were analyzed, and the occurrence of complications during the follow-up period was observed. The excellent rate of the study group patients was 83.05% (49/59), which was compared to that of the control group (81.03%, 47/58) (*P* > .05). In comparison to the control group patients, the pain, force line, and joint function scores of the study group were compared before surgery and at 3 months after surgery (*P* > .05). Compared to before surgery, the pain, force line, joint function scores, The American Orthopaedic Foot & Ankle Society (AOFAS) total score, and Berg Balance Scale (BBS) score of the patients in both groups increased at 3 months after surgery. Additionally, the AOFAS total score and BBS score of the study group patients were higher than those of the control group patients (*P* < .05). The visual analog scale (VAS) scores of the patients in the 2 groups were compared before surgery (*P* > .05). The VAS scores of the study group patients were higher than those of the control group patients at 1, 2, and 3 months after surgery (*P* < .05). The study group patients were compared with the control group patients in terms of the hallux valgus angle, the lengths of the first and second metatarsals, the angle between the first and second metatarsals, the angle of the metatarsophalangeal joint surface, the position of the sesamoids, and the shortening length of the first metatarsal (*P* > .05). The Scarf osteotomy has shown advantages in postoperative gait balance and functional scoring. Therefore, when choosing an osteotomy plan, the Scarf osteotomy can be given priority to enhance the patient’s balance ability and enhance their postoperative quality of life.

## 1. Introduction

Hallux valgus deformity is one of the common foot deformities in foot and ankle surgery. Clinically, it presents as partial dislocation of the first metatarsophalangeal joint towards the lateral side, with bony spur proliferation on the medial side of the first metatarsal head, and sesamoid deviation towards the lateral side, which can cause foot pain, discomfort, and affect wearing shoes and normal walking.^[1,2]^ For mild hallux valgus patients, corrective treatment can usually be achieved through orthopedic insoles. However, for moderate-to-severe hallux valgus patients, the corrective effects of the aforementioned conservative treatment is not satisfactory, and osteotomy surgery is usually required to correct the deformity.^[3,4]^ Currently, there are many surgical methods used clinically to treat moderate-to-severe hallux valgus deformities, each with its own advantages and disadvantages.^[5,6]^ The dual-plane osteotomy of the first metatarsal, also known as the Reverdin osteotomy combined with base osteotomy of the first metatarsal, has a good corrective effect on the deformity. However, it has a large trauma, reduces a significant amount of bone mass, and patients are prone to develop complications such as delayed healing of the osteotomy site and transfer metatarsalgia after the surgery.^[7,8]^ The Scarf osteotomy is a Z-shaped osteotomy of the first metatarsal shaft, which can correct the deformity in 3-dimensional space. The osteotomy surface is wide, which is conducive to bone healing and has good stability. However, the technique requirements for the surgeon are relatively high.^[9,10]^ This study explores the application effects and safety of the aforementioned 2 osteotomy methods for the repair and treatment of moderate-to-severe hallux valgus deformities.

## 2. Materials and methods

### 2.1. General data

This retrospective study was approved by the Ethics Committee of Shijiazhuang People’s Hospital. Acute moderate-to-severe hallux valgus patients treated at our hospital from April 2020 to April 2022 were selected. The case data of 117 patients were selected according to the inclusion and exclusion criteria for this study. Patients were divided into 2 groups according to different treatment methods. The envelope method was used to group the patients. The control group (n = 58) underwent dual-plane osteotomy of the first metatarsal, while the observation group (n = 59) underwent Scarf osteotomy. The general information of the 2 groups of patients was collected and compared (*P* > .05). See Table 1.

**Table 1:** Comparison of 2 groups of general data [ $\bar{\text{χ}}$ ± *s*,n/(%)].

Group	Age (yr)	Gender		BMI (kg/m^2^)	Other foot		Course of disease (mo)	Degree of deformity	
		Male	Female		The left	The right		Moderate	Heavy
Control group (n = 58)	45.56 ± 5.98	26 (44.83)	32 (55.17)	22.36 ± 2.11	21 (36.21)	37 (63.79)	62.33 ± 12.09	36 (62.07)	22 (37.93)
Study group (n = 59)	46.69 ± 6.11	28 (47.46)	31 (52.54)	22.51 ± 1.98	25 (42.37)	34 (57.63)	61.85 ± 11.18	38 (64.41)	21 (35.59)
χ^2^/*t*	−1.011	0.081		−0.397	0.466		0.223	0.069	
*P*	.314	.775		.692	.495		.824	.793	

Inclusion criteria: The patient was clinically diagnosed with moderate to severe hallux valgus deformity.^[11]^ The hallux valgus angle (HVA) was higher than 30°, and the intermetatarsal angle (IMA) was higher than 13°; patients were treated for the first time, no previous history of surgery; patients without other ankle deformity, such as horseshoe high arch, flat foot; all patients received conservative treatment, but no obvious effect was found.

Exclusion criteria: The patient had gout and forefoot deformity caused by rheumatoid arthritis; the lower limbs underwent major surgery in the past 3 months; patients with severe osteoporosis; patients with coagulation dysfunction; patients had corpus callosum rupture, acute and chronic infection.

### 2.2. Methods

The control group underwent dual-plane osteotomy of the first metatarsal. The patient was placed in a supine position and underwent combined spinal epidural anesthesia with lower limb nerve block anesthesia. An arterial tourniquet was placed at the root of the thigh on the affected side. A 7-cm incision was made on the medial side of the first metatarsophalangeal joint. The area was gradually dissected to expose the surgical site. A longitudinal 5-mm incision was made between the heads of the first and second metatarsals, the insertion point of the abductor hallucis muscle was cut off, the joint capsule and sesamoid suspensory ligament were loosened, the metatarsophalangeal joint was reduced, and bone spurs were removed. An oblique wedge osteotomy was performed on the medial base of the first metatarsal, and the opposite cortex was preserved. A Reverdin osteotomy was performed on the neck of the first metatarsal, and an appropriate amount of bone block was taken and implanted into the wedge osteotomy site. After shaping, a mini bone plate was used to fix the 2 osteotomy sites. An Akin osteotomy was then performed, and the medial incision was extended distally. A closed wedge osteotomy was performed on the proximal phalanx, and hollow screws were used for fixation.

The study group underwent Scarf osteotomy. The preoperative preparation, position, and anesthesia method were the same as the control group. A curved incision of approximately 5 cm was made on the dorsal medial side of the first metatarsophalangeal joint. The incision was gradually opened, and the joint capsule was exposed. A longitudinal 5 mm incision was made between the heads of the first and second metatarsals, the insertion point of the abductor hallucis muscle was cut off, and the joint capsule and sesamoid suspensory ligament were loosened. Bone spurs were removed, and a “Z”-shaped osteotomy was performed. The metatarsal head was translated and rotated according to the degree of deformity, and hollow screws were used for fixation. An Akin osteotomy was then performed, using the same method as the control group.

For patients with metastatic metatarsalgia, Weil osteotomy was performed, and routine tightening of the first metatarsophalangeal joint capsule was performed during the operation. After the surgery, patients were instructed to rest in bed and elevate the affected limb, apply local ice packs, and receive intravenous antibiotics to prevent infection. Dressing changes and suture removal were performed routinely.

### 2.3. Observation indicators

The clinical efficacy of the 2 patient groups was compared using the following criteria: Patient’s clinical efficacy was assessed based on the following parameters 3 months after surgery: hallux valgus angle (HVA) <20°, intermetatarsal angle (IMA) <9°, correction of deformity to normal, absence of bunion, absence of significant pain, normal great toe strength, and no walking difficulty as excellent; Patients who underwent X-ray examination did not demonstrate joint subluxation or dislocation and did not suffer from bunion or significant pain, with IMA measured between 10° and 20° and HVA between 20° and 25°, had mild numbness on the medial and dorsal aspects of the first toe without any other significant neurological symptoms, and had nearly normal joint mobility as good. Patients with recurrence, overcorrection, exacerbated pain, and limited joint mobility 3 months after surgery were classified as having poor outcomes. Calculating the excellent rate for both groups: ratio of sum of excellent cases to total cases.

The American Orthopaedic Foot & Ankle Society (AOFAS) score^[12]^ was used to evaluate changes in ankle function in patients before surgery and at their last follow-up visit. The score ranges from 0 to 100, with evaluations in 3 domains: alignment (0–10 points), joint function (0–40 points), and pain (0–50 points). Higher scores indicate better ankle function.

The patients’ gait balance was evaluated using the Berg Balance Scale (BBS)^[13]^ before and after surgery at their last follow-up visit. This scale consists of 14 items, each of which is scored from 0 to 4 points, with a total score ranging from 0 to 56. Higher scores on the BBS indicate better balance ability.

The patients’ postoperative pain scores were recorded at various time intervals using the visual analog scale (VAS).^[14]^ The severity of callus pain was evaluated 3 months after surgery, with a maximum score of 10 points, where 10 indicates the most severe pain.

CT scans were conducted on the patients 6 months after surgery to observe their imaging data. The following measurements were recorded for both groups: hallux valgus angle, first and second metatarsal length, first and second metatarsal interphalangeal angle, metatarsal distal articular angle, sesamoid position, and first metatarsal shortening length. The radiological results for both groups were analyzed.

The occurrence of complications during the follow-up period was recorded for each patient.

### 2.4. Statistical processing

SPSS23.0 software was used for data processing. Count data such as gender, distorted lateral foot, deformity degree, excellent and good rate were tested by chi-square test. The curative effect of patients in March after operation was tested by rank sum test, expressed as (n/%). Measurement data such as patient’s age, BMI index, AOFAS score, pain score and other groups were tested by independent sample *t* test, expressed as (‾*χ* ± *s*). In this study, α = 0.05 is the test level.

## 3. Results

### 3.1. Comparison of 2 groups of patients after 3 months of curative effect

The efficacy between the 2 groups was compared (*P* > .05). The excellent and good rate of the study group was 83.05% (49 cases/59 cases), compared with the control group (81.03%, 47 cases/58 cases; *P* > .05). See Table 2.

**Table 2:** Comparison of 2 groups of patients after 3 months of curative effect ( $\bar{\text{χ}}$ ± s, n/%).

Group	Excellent	Good	Poor	Excellent and good rate
Control group (n = 58)	24 (41.38)	23 (39.66)	11 (18.96)	47 (81.03)
Study group (n = 59)	27 (45.76)	22 (37.29)	10 (16.95)	49 (83.05)
*χ*^2^/*Z*	0.477			0.081
*P*	.633			.776

### 3.2. Comparison of AOFAS scores between the 2 groups before operation and at the last follow-up

Compared with the control group, the pain, force line and joint function scores of the study group before and 3 months after operation were compared between the 2 groups (*P* > .05). The preoperative AOFAS scores were compared between the 2 groups (*P* > .05). Compared with before operation, the pain, force line, joint function score, AOFAS total score and BBS score of the 2 groups of patients were increased at 3 months after operation, and the AOFAS total score and BBS score of the study group were higher than those of the control group (*P* < .05). See Table 3.

**Table 3:** Comparison of AOFAS scores between the 2 groups before operation and at the last follow-up ( $\bar{\text{χ}}$ ± *s*).

Group	Pain (score)		Force line (score)		Joint function (score)		AOFAS total score (score)		BBS score (score)	
	Before operation	3 mo after operation	Before operation	3 mo after operation	Before operation	3 mo after operation	Before operation	3 mo after operation	Before operation	3 mo after operation
Control group (n = 58)	21.23 ± 5.12	33.45 ± 4.46*	4.11 ± 0.64	9.45 ± 1.02*	14.77 ± 2.11	42.16 ± 3.16*	39.56 ± 4.51	81.26 ± 3.67*	18.33 ± 3.65	30.45 ± 5.21
Study group (n = 59)	21.46 ± 5.23	34.16 ± 3.98*	4.22 ± 0.55	9.66 ± 1.12*	15.02 ± 2.64	42.55 ± 2.99*	40.26 ± 3.29	83.29 ± 2.91*	18.42 ± 3.55	32.56 ± 4.09
*t*	−0.240	−0.909	−0.998	−1.060	−0.565	−0.686	−0.960	−3.318	−0.135	−2.439
*P*	.811	.365	.321	.291	.573	.494	.339	.001	.893	.016

AOFAS = The American Orthopaedic Foot & Ankle Society.

* For comparison with preoperative, *P* < .05.

### 3.3. Comparison of VAS scores between the 2 groups at different time after operation

The preoperative VAS scores of the 2 groups were compared (*P* > .05). After treatment, the VAS scores of the study group were higher than those of the control group at 1, 2 and 3 months after operation (*P* < .05). See Table 4.

**Table 4:** Comparison of VAS scores between the 2 groups at different time after operation ( $\bar{\text{χ}}$ ± *s*).

Group	Before operation	1 mo after operation	2 mo after operation	3 mo after operation
Control group (n = 58)	4.68 ± 0.56	2.16 ± 0.33	1.26 ± 0.19	0.98 ± 0.11
Study group (n = 59)	4.71 ± 0.34	3.08 ± 0.45	1.88 ± 0.45	1.31 ± 0.23
*t*	−0.351	−12.593	−9.678	−9.873
*P*	.726	<.001*	<.001*	<.001*

VAS = visual analog scale.

* Represents *P* < .001 with very significant statistical significance.

### 3.4. Comparison of imaging results between the 2 groups at 6 months after operation

The hallux valgus angle, the length of the first and second metatarsals, the angle between the first and second metatarsals, the articular surface angle of the distal metatarsal, the sesamoid position and the shortening length of the first metatarsal in the control group were compared with those in the study group (*P* > .05). See Table 5.

**Table 5:** Comparison of imaging results between the 2 groups at 6 months after operation( $\bar{\text{χ}}$ ± *s*).

Group	Foot hallux valgus angle (°)	Length of the first and second metatarsal (mm)	Angle between the first and second metatarsal (°)	Distal metatarsal articular angle (°)	Sesamoid position (grade)	The shortening length of the first metatarsal (mm)
Control group (n = 58)	13.98 ± 3.11	60.98 ± 1.98	8.11 ± 2.45	7.56 ± 1.09	2.13 ± 0.64	2.59 ± 0.48
Study group (n = 59)	14.06 ± 3.64	60.33 ± 1.89	7.98 ± 2.66	7.46 ± 1.89	2.29 ± 0.67	2.77 ± 0.64
*t*	−0.128	1.817	1.226	0.350	−1.320	−1.719
*P*	.899	.072	.223	.727	.189	.088

### 3.5. Comparison of complications between the 2 groups during follow-up

During the follow-up period, there were 1 case of delayed healing at the osteotomy site, 3 cases of deformity recurrence, 2 cases of incision infection and 2 cases of postoperative metastatic plantar pain in the study group. There were 2 cases of delayed healing at the osteotomy site, 2 cases of deformity recurrence, 3 cases of incision infection and 1 case of postoperative metastatic plantar pain in the control group. The 2 groups were compared (*P* > .05). See Table 6.

**Table 6:** Comparison of complications between the 2 groups during follow-up( $\bar{\text{χ}}$ ± *s*).

Group	Delayed healing of osteotomy site	Recurrence of deformity	Infection of incisional wound	Postoperative metastatic plantar pain
Control group (n = 58)	2 (3.45)	2 (3.45)	3 (5.17)	1 (1.72)
Study group (n = 59)	1 (1.69)	3 (5.08)	2 (3.39)	2 (3.39)
*Z*	0.436			
*P*	.663			

## 4. Discussion

Hallux valgus is a common forefoot deformity in clinical practice, with a prevalence ranging from 23% to 34%, and a higher incidence in females than males^[15,16]^ according to epidemiological studies. The mechanism of hallux valgus is complex, and previous studies have suggested that it is related to familial inheritance, poor footwear habits, and other factors.^[17,18]^ Severe pain caused by moderate to severe hallux valgus often requires surgical correction. Currently, there are over 100 surgical methods used in the clinical treatment of hallux valgus deformities. Common osteotomy procedures include the first metatarsal base osteotomy combined with Reverdin osteotomy double flat osteotomy and Scarf osteotomy, among others.^[19,20]^ Studies have shown that double flat osteotomy has a strong corrective effect on hallux valgus and is particularly effective for patients with a larger metatarsal distal articular angle. However, it is associated with a larger trauma and relatively poor stability.^[21]^ On the other hand, the Scarf osteotomy provides good stability but may result in overlapping of the bones and requires higher surgical skills.^[22]^

This study compared the therapeutic effects of double flat osteotomy and Scarf osteotomy in the treatment of moderate to severe hallux valgus and found no statistically significant difference in the therapeutic effectiveness between the 2 groups. There were no statistically significant differences observed in the foot hallux valgus angle, first and second metatarsal length, first and second metatarsal intermetatarsal angle, metatarsal distal articular angle, sesamoid position, and first metatarsal shortening length between the 2 groups of patients. These results suggest that both Scarf osteotomy and first metatarsal double flat osteotomy are effective in correcting hallux valgus deformities with similar clinical outcomes. The double flat osteotomy of the first metatarsal involves 2 osteotomies performed on the medial and neck regions of the first metatarsal to achieve better correction of hallux valgus deformities.^[23,24]^ The Scarf osteotomy involves a “Z”-shaped osteotomy of the first metatarsal, followed by distal translation and rotation of the bone, which can effectively correct hallux valgus and restore the biomechanical alignment of the forefoot.^[25,26]^

This study found that compared with preoperative measurements, in both groups of patients, the pain level, alignment, joint function scores, AOFAS total scores, and BBS scores were all improved at 3 months after treatment. Additionally, the AOFAS total scores and BBS scores of the study group were higher than those of the control group. However, the VAS scores of the study group at 1, 2, and 3 months after surgery were higher than those of the control group. The above results suggest that the Scarf osteotomy has certain advantages in postoperative gait balance and functional scores, but it causes more severe pain after surgery than the double flat osteotomy. This is because the Scarf osteotomy involves a multi-planar “Z”-shaped osteotomy of the metatarsal shaft, which can correct hallux valgus by moving the bone block laterally. It provides greater stability and allows patients to get out of bed and move earlier with the assistance of a non-weight-bearing shoe after surgery. As a result, postoperative gait balance and function can be better restored.^[27,28]^ However, the Scarf osteotomy is prone to have “stair-step” effect, which can cause elevation of the first metatarsal and transfer pain. The double flat osteotomy involves removing more bone and has a larger incision, which may affect the patient’s postoperative gait balance. However, this procedure can change the shape of the first metatarsal to share the load of the second metatarsal, causing the head of the first metatarsal to sink, thereby causing less severe pain.^[29,30]^

Some studies suggest that compared with the Scarf osteotomy, the double flat osteotomy has a larger incision and requires more stripping of soft tissue and bone membrane during the procedure, which causes greater trauma and is more prone to postoperative complications.^[31–33]^ This study found that the main postoperative complications in both groups were delayed healing at the osteotomy site, relapse of deformity, incision infection, and transfer pain. There was no significant difference in the incidence of postoperative complications between the 2 groups. This result suggests that the risks of complications caused by Scarf osteotomy and double flat osteotomy of the first metatarsal are similar. This finding is inconsistent with the conclusions of previous clinical studies.^[34,35]^ This may be due to the bias caused by the small sample size of this study. In future clinical work, a larger sample size should be accumulated to further explore whether Scarf osteotomy has an advantage in reducing postoperative complications.

Based on the findings of this study, clinicians should consider the Scarf osteotomy as a primary surgical option for patients with moderate to severe hallux valgus deformities, especially for those who prioritize functional recovery and balance improvement. Training and proficiency in the Scarf osteotomy technique are recommended to maximize its benefits and minimize potential complications. Furthermore, patient education on postoperative rehabilitation protocols can enhance the functional outcomes associated with this procedure.

In summary, there is no significant difference in clinical efficacy between Scarf osteotomy and double flat osteotomy of the first metatarsal in the treatment of moderate to severe hallux valgus deformity. However, Scarf osteotomy has advantages in postoperative gait balance and functional scores. Therefore, Scarf osteotomy can be considered as the preferred osteotomy option to enhance patients’ balance ability and enhance postoperative quality of life.

## Author contributions

**Conceptualization:** Zhe Xie, Zhaona Wang, Shan Zhai, Lixing Zhang, Yanbing Gao, Hailong Wu.

**Data curation:** Zhe Xie, Zhaona Wang, Shan Zhai, Jianzhong Wu, Lixing Zhang.

**Formal analysis:** Shan Zhai, Yanbing Gao, Hailong Wu.

**Funding acquisition:** Zhaona Wang, Hailong Wu.

**Investigation:** Zhaona Wang, Shan Zhai, Jianzhong Wu, Lixing Zhang, Yanbing Gao, Hailong Wu.

**Methodology:** Zhaona Wang, Jianzhong Wu, Hailong Wu.

**Supervision:** Jianzhong Wu, Lixing Zhang, Yanbing Gao, Hailong Wu.

**Validation:** Lixing Zhang.

**Writing – original draft:** Zhe Xie, Zhaona Wang, Hailong Wu.

**Writing – review & editing:** Zhe Xie, Hailong Wu.
